# Manipulation and Mixing of 200 Femtoliter Droplets in Nanofluidic Channels Using MHz‐Order Surface Acoustic Waves

**DOI:** 10.1002/advs.202100408

**Published:** 2021-05-16

**Authors:** Naiqing Zhang, Amihai Horesh, James Friend

**Affiliations:** ^1^ Medically Advanced Devices Lab, Center for Medical Devices, Department of Mechanical and Aerospace Engineering, Jacobs School of Engineering and Department of Surgery, School of Medicine, 9500 Gilman Dr. MC0411 University of California San Diego La Jolla CA 92093 USA

**Keywords:** acoustofluidics, digital fluidics, droplets, nanofluidics, surface acoustic wave

## Abstract

Controllable manipulation and effective mixing of fluids and colloids at the nanoscale is made exceptionally difficult by the dominance of surface and viscous forces. The use of megahertz (MHz)‐order vibration has dramatically expanded in microfluidics, enabling fluid manipulation, atomization, and microscale particle and cell separation. Even more powerful results are found at the nanoscale, with the key discovery of new regimes of acoustic wave interaction with 200 fL droplets of deionized water. It is shown that 40 MHz‐order surface acoustic waves can manipulate such droplets within fully transparent, high‐aspect ratio, 100 nm tall, 20–130 micron wide, 5‐mm long nanoslit channels. By forming traps as locally widened regions along such a channel, individual fluid droplets may be propelled from one trap to the next, split between them, mixed, and merged. A simple theory is provided to describe the mechanisms of droplet transport and splitting.

## Graphical abstract

Digital nanofluidics is enabled through rapid transport, splitting, combination, and mixing of 200 fL droplets using surface acoustic waves in a specially shaped, 130–150‐nm tall nanoslit channel with discretely entrapped droplets.

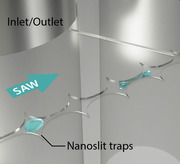

## 1 Introduction

Nanofluidics^[^ ^1^ ^]^ has been proposed as a useful means to biological analysis and sensing,^[^ ^2^ ^]^ medical diagnosis,^[^ ^3^ ^]^ and material processing.^[^ ^4^ ^]^ The analytical devices devised to work at such small scales employ nanoliter to picoliter fluid volumes, surface area‐to‐volume ratios of ∼10^7^ m^−1^ and more, and the minimum feature sizes that are possible to fabricate using massively parallelized, top‐down fabrication technology.^[^ ^5^, ^6^, ^7^ ^]^ A notable example is the scaling of liquid chromatography down to the nanoscale, where femtoliter (fL) to attoliter samples have been injected and successfully separated, producing vastly shorter separation times—to a few seconds, and higher separation resolution—to 7 000 000 plates m^−1^.^[^ ^8^ ^]^

The idea of manipulating droplets in nanofluidics devices is enticing, as the volume of such droplets—from picoliter to attoliter^[^ ^9^ ^]^—approaches the size of individual large molecules and nano‐objects. Consequently, it may potentially revolutionize medical diagnostics and personalized treatment^[^ ^3^ ^]^ by increasing the sensitivity of the analytical tools underpinning these disciplines.^[^ ^10^, ^11^ ^]^ Manipulation of these droplets is crucial for these applications and beyond small‐volume biological integration and analysis for single molecule‐in‐cell applications.^[^ ^7^, ^12^, ^13^ ^]^

However, manipulation of fluids and colloids at the nanoscale is made exceptionally difficult by the dominance of surface and viscous forces. Consider a typical water droplet entrapped in an air‐filled, 10 × 10 µm square cross‐section microchannel. The capillary (Laplace) pressure Δ*P* = γ(1/*R*_1_ + 1/*R*_2_) ≈ 14.5 kPa, where γ_water_ = 72.3 mN m^−1^ is the surface tension for water and *R*_1_ and *R*_2_ are the radii of curvatures. Reducing that channel to a 10 × 10 nm nanochannel increases the capillary pressure to 14.5 MPa, 145 times atmospheric pressure at sea level, and very difficult to overcome by any means. Achieving this and transporting such a droplet through a nanochannel is both a significant challenge and crucial to achieving the promise of nanofluidics.

For this purpose, a variety of pump designs that curiously employ carbon nanotubes have been proposed over the past 15 years, using temperature gradients,^[^ ^14^, ^15^ ^]^ Coulomb drag,^[^ ^16^ ^]^ surface waves (in theory),^[^ ^17^ ^]^ or static electric fields.^[^ ^18^, ^19^, ^20^, ^21^ ^]^ Passive fL‐scale fluid handling has also been devised using surface modification or geometric channel design.^[^ ^7^, ^13^ ^]^ However, despite all these attempts, due to the overwhelming dominance of surface tension, no effective active manipulation method has been experimentally demonstrated in a nanoscale enclosed fluidic channel system to date to our knowledge.

The use of MHz‐order vibration has dramatically expanded in microfluidics, introducing acoustic streaming^[^ ^22^ ^]^ and enabling fluid manipulation,^[^ ^23^, ^24^, ^25^ ^]^ atomization,^[^ ^26^, ^27^ ^]^ and microscale to nanoscale particle/cell separation.^[^ ^28^, ^29^, ^30^, ^31^ ^]^ A novel surface acoustic wave (SAW) induced pumping method has been reported to manipulate nanoslit channel‐confined fluids and suspended nanoparticles and molecules.^[^ ^32^ ^]^ We find even more powerful results at the nanoscale, with the key discovery of a new mechanism of acoustic wave‐fluid motion interaction.^[^ ^33^ ^]^ It arises from nonlinear interactions between the surrounding channel deformation and the leading order acoustic pressure field, generating flow pressures three orders of magnitude greater than any known acoustically‐mediated mechanism.

Transmitted along the length of a nanochannel, SAW was shown to be capable of delivering large pressures and rapid flows sufficient to overcome surface‐mediated forces and produce fluid transport. While the production of continuous flow of fluids and colloids of particles and molecules in nanoslit channels is interesting, there may be the opportunity to exploit SAW to manipulate discrete droplets in nanoscale channels—a form of digital nanofluidics. Such confined droplets would have femtoliter to attoliter volumes, a promising result in seeking to work with extremely small quantities of analytes.

**Figure 1:**
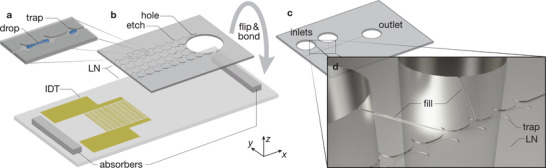
Concept and fabrication of SAW‐integrated nanofluidic femtoliter droplet devices. a) A nonuniform nanoslit channel produces fluid droplet traps. b) By etching several of these channels into an LN layer, cutting a 1 mm hole at the end distant from the SAW interdigital transducer (IDT), and then flipping the result and bonding it with room‐temperature LN‐LN bonding,^[^ ^34^ ^]^ machined‐side down onto the LN substrate that has the SAW IDT, it is possible to form and transport drops in these channels with heights to less than 10 nm (figures not to scale for clarity). However, a ∼100 nm height was chosen to obtain the best possible performance of the acoustic wave propagation in the nanoslit using acoustogeometric streaming.^[^ ^33^ ^]^ Absorbers placed at the SAW LN device's ends absorb extraneous SAW and prevents undesirable reflections. c) Other configurations make it possible to explore drop splitting, mixing, and transport, such as this configuration with only one main channel of ten traps, two of which are connected to inlets at the side, and the *x*‐axis‐oriented main channel is open to the outside close to the IDT while it is connected to an outlet at the distant end. d) The inlets are connected to individual, adjacent traps in this configuration by 10 µm wide channels designed to slowly carry fluid to the main channel.

Here, we present active 200‐fL fluid droplet manipulation using MHz‐order SAW within high‐aspect ratio, 100‐nm tall, 20–130‐µm wide, 5‐mm long nanoslit channels, fabricated via a direct, room‐temperature bonding method^[^ ^34^ ^]^ for lithium niobate (LN) as illustrated in **Figure** **1**. The undulating shape of wide traps connected by narrow necks was intentionally chosen to trap droplets in discrete locations and facilitate their manipulation among these traps. Contrary to the results seen in the past,^[^ ^32^ ^]^ a droplet will locate at a point where its surface energy is lower, the wider region of the trap in our system. The dimensions of the traps were chosen to entrap 200‐fL droplets, which are 50 µm in diameter in a 100‐nm tall channel. The radius of curvature of each of the four walls that form the trap was 60 µm, slightly larger than the trapped droplet, to ensure its central location while maintaining separation between the wall and fluid. The curvature of this shape was chosen to eliminate corners that could capture and trap droplets. These walls naturally lead to another trap via extension of one of the vertices of the trap to form a “neck” between them. The width of the neck was chosen to be 20 µm, less than half the diameter of the 200‐fL droplets but large enough to reliably fabricate in our process. Feature sizes of 2 µm may be fabricated, but the yield is better when choosing feature sizes greater than about 10 µm.

It is possible to induce ≈200‐fL droplet splitting and transport, phenomena that are governed by the time and power of the SAW actuation for a given fluid. We provide a closed‐form analytical model that accurately describes the boundaries between these handling phenomena. We also report the ability to induce merging and mixing in a ≈200‐fL droplet, operations desirable in fluid handling whatever the scale. As an exploratory work, we set aside the issue of evaporation, actually exploiting it in forming droplets for this study. Evaporation is nonetheless an important constraint in droplet nanofluidics, more so than in droplet‐based digital microfluidics, and the methods to overcome it at the microscale^[^ ^35^, ^36^ ^]^ may prove beneficial at the nanoscale. Altogether, our results indicate MHz‐order SAW is a powerful tool for discrete fluid droplet manipulation at the nanoscale.

## 2 Results and Discussion

We designed an acoustic nanofluidic system in which the enclosed channel height was chosen to match, approximately, the viscous penetration depth of about 100 nm. This choice is based on some details of the acousto‐geometric streaming phenomena that is unique to nanochannels when using ∼10 MHz acoustic waves. Specifically, we rely upon the physical deformation of the channel walls in direct, nonlinear coupling with the acoustic field present in the fluid to produce rapid flow against a very large pressure head.^[^ ^33^ ^]^ This is rather different than other, traditional forms of acoustic streaming which relies on compressibility of the fluid, and which are, by comparison, exceedingly weak and would never work at the nanoscale. How this new form of acoustic streaming will act upon droplets in such a device is unclear, a key motivation for this study.

We have produced a model of the phenomena based upon an energy balance between acoustic and capillary phenomena. Details of this model along with the requisite definitions of the symbols are provided in Section 4. The model has no unknown constants and is completely independent of the experimental results.

### 2.1 Observations of Femtoliter Droplet Manipulation

**Figure 2:**
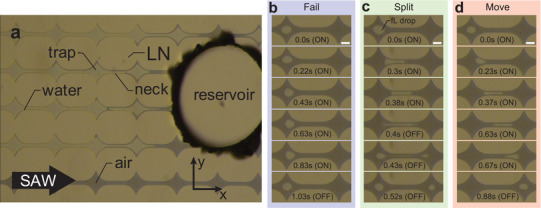
a) The 100 nm height nanochannel matrix, with traps as shown separated by narrow regions, or necks. The channels are filled with DI water from the reservoir at right. SAW‐driven evaporation slowly eliminates the water from the nanochannels over a period of about 1 min. The experiment begins when the droplet diameter is 50 µm, corresponding to a volume of 200 fL. For scale, the narrowest width of the neck halfway between the traps is 20 µm and the trap is 130 µm at its widest in all figures in this study. The dark areas indicate trapped air and light areas are entrapped water. The direction of the SAW is from left to right. With judicious filling and evaporation, it is possible to form b) a single droplet within a trap. After applying SAW, the (b‐d) image sequences indicate three possible outcomes. Application of 270 mW SAW at 40 MHz for an activation time of 1.03 s (b) fails to propel a 200 fL droplet from one trap to the next. Capillary force overcomes the propulsive acoustic force. If, however, the SAW power is greater at 1.3 W for only 0.38 s, the droplet will (c) split. The droplet becomes trapped in the neck and a portion escapes to the next trap and the remainder returns to the original trap. With a longer SAW activation time of 0.67 s at the same power of 1.3 W, the SAW (d) completely moves the droplet to the next trap. Scale bars: 40 µm.

After judiciously filling the nanoslit channel structure with DI water (**Figure** **2**a), SAW‐driven evaporation at an input power of 0.1 W is used to form a droplet in a single trap (e.g., the droplet in Figure 2b–d). The evaporation proceeds over ≈1 min to produce a droplet diameter of 50 µm, representing a 200 fL droplet in this system. Without the application of SAW, the evaporation is much slower, taking about 20 min to evaporate a 200 fL droplet of water at one of the traps. Because the manipulation of the droplets we report in this study occurs quickly, much faster than 1 min, and because the anticipated applications of this technology are in devices that would need to produce results in a few minutes at most, we are able to proceed.

It becomes possible to (Figure 2c,d) transport the droplet to the next trap using SAW, along the direction of the SAW's propagation. From trap to trap, the droplet must deform to pass through the neck between them, increasing its surface area and consequently its surface energy. The necks between traps, therefore, represent regions of high droplet surface energy between the lower droplet surface energy required when it is in one of the traps. With deionized (DI) water, the pressure difference from trap to neck is ∼1 kPa. While this capillary pressure is still greater than what could realistically be generated from traditional acoustic streaming, it is still three orders of magnitude less than the ∼1 MPa capillary pressure of water in filling the nanoslit channel from the outside. Lithium niobate is hydrophilic with a contact angle^[^ ^37^ ^]^ of about 25°, so channels formed of LN tend to easily fill.

Returning now to the behavior of discrete droplets in the nanoslit channel, we find three distinct behaviors depending on the input power and duration of the activation time for the SAW. Insufficient input energy causes a failure to transport the droplet from one trap to the next, illustrated in Figure 2b. A small amount of motion is observed, but the droplet remains in the same trap. With a greater amount of energy, the droplet will partially enter the neck between the two traps before splitting (see Figure 2c). With sufficient input energy, however, the droplet may be completely moved to the next trap as in Figure 2d.

**Figure 3:**
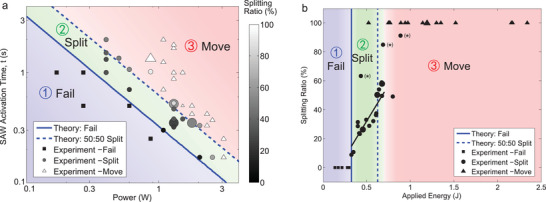
An analytical model suggests three regimes for droplet transport in the nanoslit device between traps, with comparison of experimental results using DI water with a surface tension of 72 mN m^−1^. a) The analytical and experimental results are log‐log plotted as SAW activation time versus applied power, showing that the independent experimental results (with 43 test samples in total) are correlated with the model. The results are provided (b) again, this time in terms of the splitting ratio versus applied energy, the product of (a) SAW power and activation time. The droplet transport behavior is mainly dependent on the applied acoustic energy, with a narrow region (2) of splitting between (1) failure to transport and (3) complete movement of the droplet to the next trap. Note that successful splitting occurs in delivering 10–60% of the parent droplet's volume to the new trap. It is generally difficult to split more than 60% of the parent droplet's volume and transport it to the new trap, with only three successful (*) examples out of 35 trials for these specific conditions. The reason this occurs is explained and discussed in Section 4.4. Omitting these three points, a linear fit of the remaining splitting droplet data (with 18 test samples in total) in region 2 (black line) indicates a reasonable correlation (*R* ^2^ = 0.779) between the applied input energy and the volume of the split droplet present in the new trap. A linear plot of the (a) SAW activation time versus power is provided in Figure S1, Supporting Information, to indicate the close correlation of the theory with experiments in DI water and isopropyl alcohol. Video footage of the droplet behaviors along with details of how the droplet volume is calculated from observations are also described in the Supporting Information. Symbol sizes here represent the droplet volume; most are a medium size for ≈200 fL, while a few are large for ≈400 fL and small for ≈100 fL. The effect of the droplet size is weak. The data was confirmed to be normally distributed via a Shapiro–Wilk test, and the text describes the outcomes from a test of monotonicity in region 2 using Spearman's rank coefficient.

An independent analytical model, detailed in Section 4, suggests three regimes for droplet transport in the nanoslit device between traps in **Figure** **3**. The results from that model are plotted here using DI water with a surface tension of γ = 72 mN/m. Region 1 represents a failure to transport any or all of the droplet's volume from a trap. This occurs when the product of the applied SAW power and its duration, the input energy, is below a critical value, (*C*_1_ − *C*_0_)*h*γ, defined mainly by the nanoslit height, *h* and the surface tension of the fluid, γ. Taken from Section 4, this represents a droplet surface energy difference, from the trap to the neck, to be overcome by the input energy. The constant (*C*_1_ − *C*_0_) represents the change in the circumference of the droplet as it moves from the trap to the neck, with the right end of the drop at the midpoint or narrowest point of the neck. The critical energy demarcating failure (region 1) from other phenomena (regions 2 and 3) is plotted with a thick blue line in Figure 3.

As the power is increased, the time required to at least obtain splitting is decreased. In our experiments, we used at least 100 mW of input power, the lower limit in our system while avoiding undue signal noise; at that power about 2 s or more would be required according to the theory. In the DI water experiments, using 200–300 mW for 1 s failed to transport the droplet. At 2 W and 0.2 s, the applied SAW split the droplet trapped in the first trap, but only delivered about 20% to the next trap. Increasing the power for this same time of application to 2.5 W delivers 50% to the next trap, and 3 W moves the entire droplet. In this system, 3 W represented the maximum power we were able to use, limited by atomization or jetting^[^ ^38^ ^]^ and other curious effects outside the scope of this work.

There is an intermediate range of SAW energy at which it is possible to partially carry the droplet through the neck to the next trap in region 2, splitting the droplet within the neck, and causing some of the volume to return to the original trap and the remainder traveling onward to the next trap. A second, dashed line is plotted from analysis results in Figure 3 to indicate equal (50:50) splitting of the droplet between the traps. The necessary deformation from a circular cylindrical droplet in the trap to a thin elliptical droplet in the neck symmetrically placed between two traps is conceptualized in Figure 1a.

In the experiments, it was observed that it is possible to split between 10% and 60% of the parent droplet's volume and transport this to the next trap. It was generally difficult to split and transport more than 60% of the parent droplet's volume, as discussed in more detail in Section 4.4. With very few exceptions (as indicated with asterisks in Figure 3b), in seeking to move such a large portion of the parent droplet to the new trap, generally the entire droplet was moved instead. Omitting these three points, a linear fit of the remaining splitting droplet data in region 2 (black line, Figure 3b) indicates a reasonable correlation (*R* ^2^ = 0.779) between the applied input energy and the volume of the split droplet present in the new trap. This is expected as the droplet requires progressively larger amounts of energy to deform it into the neck until it reaches the 50:50 split location. It is also supported via a Spearman's rank correlation coefficient computation, producing *r* = 0.901 with a 95% confidence interval from 0.744 to 0.964, a nearly perfect increasing monotonicity of splitting ratio as a function of input energy in this regime.

Likewise, it was difficult to split less than about 10% of the droplet volume off from the parent droplet to be transported to the new trap. It is suspected that if the SAW was applied with greater precision in power and time, droplet volumes smaller than 10% could possibly be split and transported to the new trap. However, the lateral size of a droplet entrapped in the nanoslit with a volume of 20 fL, 10% of the parent 200 fL droplet, is only 8 µm, less than half of the neck width: 20 µm. Droplets smaller than 20 fL are suspected to be difficult to produce unless the neck width is also made smaller to produce greater lateral confinement and deformation of the parent drop.

If the SAW power and duration, and therefore the input energy, are even greater, the droplet will be entirely transported to the next trap as defined by region 3. The additional energy required is the kinetic energy necessary to move the majority—at least 60%—of the droplet beyond the middle of the neck. The vagueness here is due in part to the difficulty in pinching off and splitting less than 40% of the trailing portion of the droplet to return to the original trap. It is also due to the relatively slow capillary time in comparison to the time required for the droplet to traverse the neck to the next trap, detailed in Section 4.

Nonetheless, this model accurately describes the three observed droplet manipulation regions in Figure S1, Supporting Information, for this and isopropyl alcohol as an example of another fluid. Video footage of the droplet behaviors is provided in the Supporting Information for additional context.

In our device, the acoustic energy imparted into the droplet is $E_{\text{ac}}=\frac{A}{w\alpha^{-1}}\eta W\left(1-1/e\right)t\approx \;10^{-11}$ J.^[^ ^39^ ^]^ This is on the same order as the change in capillary energy required to move the droplet from a circular cylindrical shape (circumference *C*_0_) in the trap to the narrow elliptical shape (circumference *C*_2_) in the neck, *E*_cap_ = (*C*_2_ − *C*_0_)*h*γ ≈ 10^−11^ J. The fact the acoustic and surface energies are on the same order supports the notion of using SAW to transport these droplets from trap to neck—and onward to the next trap.

### 2.2 Femtoliter Droplet Merging and Mixing

#### 2.2.1 Merging Droplets at the Nanoscale

**Figure 4:**
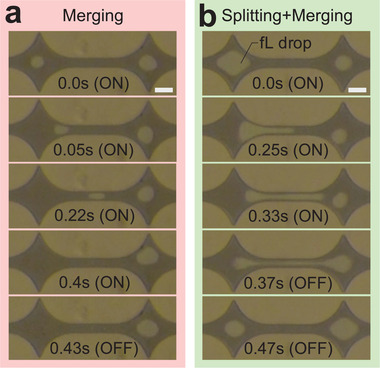
Image series showing femtoliter droplets merging via SAW actuation in a ∼100 nm‐height nanochannel. The SAW propagates from left to right. a) The droplet in the left trap is completely transported and merged with the droplet in the right trap using a SAW power of 1.78 W and activation time of 0.42 s. b) The droplet in the left trap is split with the same SAW power of 1.78 W but a shorter activation time of 0.35 s. The split portion is merged with the droplet present in the right trap; the remainder returns to the left trap. Adjusting the SAW power and activation time controls the splitting and merging phenomenon. Scale bar: 40 µm.

After introducing SAW‐induced fluid manipulation of a single droplet in a nanoscale channel, here we present the merging of two femtoliter droplets in a nanochannel using SAW. We are able to partially (split) or completely (move) transport one femtoliter droplet to the next trap, merging it with a droplet already located there. **Figure** **4** provides two examples of the merging phenomenon while using 1.78 W SAW power. Figure 4a demonstrates an entire droplet transported from one trap to the next to merge it with a second droplet. Figure 4b shows the splitting of the parent droplet in the trap at left, followed by the merging of the portion that travels through the neck to the droplet already present there. The key difference between the two results was the use of a different SAW activation time, 0.42 s for the a) entire droplet, and 0.35 s for b) splitting and merging, respectively.

The presence of the droplet at left in these examples acts to absorb a significant amount of SAW propagating from left to right in Figure 4. The remaining SAW energy, if any, that interacts with the droplet at the right is insufficient to move or split it.

This produces another useful tool in droplet manipulation at these small scales. A careful look at Figure 4 shows that the starting volume of the droplets is slightly different. This only weakly affects the splitting and moving behavior in this system.

Using the analysis from Section 4, the starting circumference of a larger droplet is itself larger. Therefore, the increment in energy required to place it into the neck might at first seem to be lower, because the starting free surface energy is larger. However, a larger droplet must also have the same semiminor axis length that matches the neck width while extending farther along the channel to produce a larger semimajor axis length. The energy required to put it in the middle of the neck for a 50:50 split, for example, is therefore also greater. These two effects are approximately the same, producing approximately the same result for different starting droplet volumes.

Whether for transport, splitting, or merging, the effect of the droplet size appears to be weak between 100 and 400 fL, because the basic shape undertaken by the droplet when in the trap and in the neck between the traps is similar to a 200 fL droplet. The SAW is pervasive, present everywhere, and changing the miniscule size of these droplets has no observable effect on the SAW nor the interaction between the droplet and SAW. At less than 100 fL, the “pinching” effect of the neck is eliminated, implying droplets smaller in diameter than about 20 µm in the system will simply pass through the neck from trap to trap without any change in surface energy. Beyond 400 fL, the shape of the entrapped droplet is significantly affected by the trap shape. These effects are only due to the design, and, for example, a narrower neck combined with larger traps may improve the range of fluid volumes. However, such changes may cause other difficulties, including less ability to define the droplet size via evaporation and significantly greater time to transport, split, or merge the droplets.

There is also the matter of the product of input SAW power, *W* and its active duration, *t* producing a given input SAW acoustic energy *E*_ac, in_ = *Wt*. In manipulating single droplets, it was apparent from Figure 3 that the SAW acoustic energy alone was sufficient to describe the droplet behavior. With a pair of droplets, we have observed that the merging results—whether splitting and merging or merging alone—are generally improved for a given acoustic energy by increasing the power and decreasing the activation time.

The difference in SAW power upon the left and right droplets in their respective traps is greater as the input power increases. There is no effect of changing the time, as it is always longer than the time it takes for the SAW to propagate throughout the device. Therefore, using a greater power for a shorter time produces a greater difference in acoustic energy applied to the two droplets, and this is why the results improve.

There is an opportunity for substantial further work here, by employing different fluids, different trap‐neck‐trap geometries, the SAW characteristics including its frequency and modulation, programmable acoustic actuation for two‐dimensional droplet manipulation, and certainly extension to practical experiments in biology and chemistry. Our main purpose was to show the existence of the basic tools to facilitate these efforts, and we now add another useful tool: mixing.

#### 2.2.2 Mixing Within Single Femtoliter Droplets Using SAW

Mixing has long been a desirable outcome in sample processing and chemistry whatever the scale of the sample, and is a challenge at the micro‐scale and beyond to smaller dimensions. Even though the droplet is small, it is confined, and diffusion is slow. The characteristic time of diffusion is *t*_d_ ≈ *x* ^2^/*D* ≈ 10^2^/10^1^ s = 10 s for a typical nanoparticle in water,^[^ ^40^ ^]^ though at these scales the surface chemistry of the particle can significantly affect the diffusion rate.

Nonetheless, it would still be beneficial to speed the mixing process if possible in such a droplet through convection. Here we demonstrate SAW‐induced convective mixing within a single 400 fL droplet while it remains stationary in a trap, using 50 nm nanoparticles. This is accomplished by exposing it to SAW energy less than the threshold defined in Figure 3 between regions 1 and 2.

**Figure 5:**
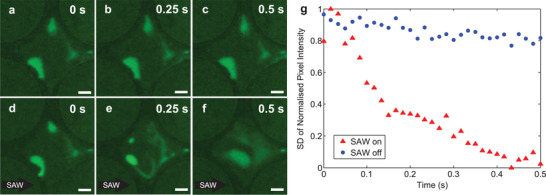
Effective SAW induced fluid mixing of 50 nm green fluorescent polystyrene particles within a 400 fL droplet in an *h* = 100 nm nanoslit trap. (a–c) Images of the trapped, nanoparticle‐laden droplet in the trap without SAW at 0, 0.25, and 0.5 s, showing negligible mixing with nearly identical images. However, when using 380 mW SAW (left to right in images), (d–f) mixing occurs to produce a comparatively complete mixing result over the same time period. Scale bars: 20 µm. A plot of the g) standard deviation (SD) of the normalized pixel intensity from these and additional images in the sequence (with 31 test samples over 0.5 s from images captured at 60 fps) with and without SAW serve to quantify the effectiveness of the mixing. With SAW, the mixing is nearly complete in 0.4 s, with SD → 0 in that time. By comparison, without SAW, the SD declines from about 1 to 0.85 over 0.5 s. The pixel intensity data was confirmed to be normal via the Shapiro‐Wilk test and the monotonic decrease for each data set was confirmed by computing Spearman's rank correlation coefficient: *r* = −0.982 while the SAW was on and *r* = −0.814 while the SAW was off.

To conduct this experiment, DI water mixed with 50 nm nanoparticles at a concentration of 3.64 × 10^11^ particles per µL were delivered into the system shown in Figure 2a, filling the nanoslit channels via capillary wetting. Evaporation was allowed to proceed for about 30 s to produce the result in **Figure** **5**a, a distinctly separated and locally concentrated suspension of nanoparticles within a fluid droplet of ≈400 fL filling one of the traps of the device. Over time, diffusion only weakly affects the distribution of particles (Figure 5a–c) without SAW. However, introducing 380 mW SAW for 0.5 s, notably in region 1 of Figure 3, vortices generated by apparent acoustic streaming within the droplet cause the rapid mixing of the suspended nanoparticles to produce a relatively well‐mixed result in 0.5 s (Figure 5d–f).

This result may be quantified^[^ ^41^ ^]^ by calculating the standard deviation of the pixel intensity of the captured images in Figure 5a–f. If the particles remain unmixed and locally concentrated, they produce local regions of brighter fluorescence and intensity, surrounded by relatively dark regions where the particles are few in number. This produces a large range of pixel intensity, likewise producing a large standard deviation. The result for the diffusion‐only nanoparticle mixing process is compared to the SAW‐aided mixing in Figure 5g. Notably, both begin with a similar standard deviation at one, reflecting the range of pixel intensities at this time from zero to one. Diffusion alone gradually reduces the standard deviation to 0.85 after 0.5 s. However, with SAW, the standard deviation goes to zero, indicating complete mixing, in just over 0.4 s. Spearman's rank correlation coefficients (*r*) were computed for each case to evaluate both the monotonic decrease in the standard deviation with respect to time and the effect of using SAW. The coefficient *r* = −0.982 while SAW was on with a 95% confidence interval from −0.992 to −0.962, and *r* = −0.814 while the SAW was off with a 95% confidence interval from −0.909 to −0.640. This indicates a stronger decreasing monotonic trend of the standard deviation with respect to time with SAW actuation than with thermal diffusion alone.

While most chemical processes are of course molecules, and therefore far smaller and far more rapidly diffusing than nanoparticles, the ability to mix is still believed to be beneficial in significantly speeding up the process. In Figure 5g, the initial reduction in the standard deviation is very rapid, from 1 to 0.4 in 0.15 s, a consequence of most of the droplet mixing. A small region at lower left seen in Figure 5d,e takes longer to mix, contributing to the slower result seen from 0.15 to 0.4 s. This is believed to be due to the fact that this portion of the droplet is not located in the main channel of the nanoslit, and the SAW is at a lower magnitude here. It may be possible to produce even more rapid mixing results if the trap is redesigned to also accommodate mixing and prevent these regions.

In this system, generally the viscous penetration depth δ ≈ 100 nm is nearly equal to the channel height. Thus, the mixing seen here is mixing occurring in the viscous boundary layer. The flow patterns that appear are defined by the droplet size. In other words, there are no apparent internal vortices or mixing at shorter length scales. If the droplets were larger, it may be possible that the mixing occurs on the same scale as the wavelength of the SAW, based on past observations.^[^ ^32^, ^33^ ^]^ However, the wavelength of the 40 MHz SAW used here is 100 µm, while the (lateral) droplet size is also about 100 µm.

## 3 Conclusions

We developed a simple technique for femtoliter droplet manipulation in a nanofluidic system using high frequency SAW. Nano‐height channels were fabricated using reactive ion etching and room temperature LN–LN bonding.

We focused on water in our experiments, as most applications would employ water. We also provide results using isopropyl alcohol in the Supporting Information, showing similarly good correlation between experiment and analysis. Switching fluids at these small scales is more problematic than at the micro or larger scales. Initially, the femtoliter droplets are located in the wider region of the nanochannels—traps—where their surface free energy is minimized. But by applying SAW, it is possible to introduce sufficient energy to permit them to be deformed and pass through the narrow necks between traps to facilitate drop splitting and transport.

A model of the phenomena was devised, and since the model has no constants to adjust, it is reasonable to conclude the simple models adequately represent the observed phenomena, based on the close correlations between the experimental results and the theoretical model. We also demonstrated the ability to merge and rapidly mix droplets in the system, additional useful tools for future digital nanofluidics.

These results suggest ideas for future changes in the channel profile to produce better droplet handling outcomes as desired when manipulating them via SAW, for example, to make it possible to manipulate a greater range of droplet sizes, to tailor the design to split extremely small portions of fluid from a parent drop, and to expand these devices to perform in a parallel fashion with many droplets at once, both laterally and axially. Furthermore, given the rapid response of SAW, it may be possible to perform more complex manipulations by modulating the SAW over time. Finally, by combining the various operations reported in this paper with novel droplet generation methods under study by other groups,^[^ ^42^ ^]^ SAW nanofluidics may become far more straightforward to use in applications, providing an extraordinary opportunity to achieve the lofty aims of “lab‐on‐a‐chip” via useful nanofluidics.

## 4 Experimental Section

##### Device and Nanoslit Channel Fabrication

An acoustic nanofluidic system was designed in which the viscous penetration depth was approximately identical to the characteristic height of the channel (i.e., ≈100 nm). Fabrication of nanoheight channels was performed incorporating surface acoustic wave actuation on 128° Y‐rotated X‐propagating lithium niobate (LN, Precision Micro‐Optics Inc., Burlington, MA, USA) substrate.^[^ ^32^, ^34^ ^]^ Reactive ion etching (Plasmalab 100, Oxford Instruments, Abingdon, UK) was first utilized to create a nanoheight depression of the undulating channels (130 µm at its widest and 20 µm at its most narrow) on an LN substrate with patterned Cr as a sacrificial mask. The Cr sacrificial mask was later removed by Cr etchant (Figure 1(a1)).

A 1‐mm hole was then drilled (Dremel #4000, Mount Prospect, Illinois, USA) through the chip to form an input liquid reservoir for the nanoslit system (Figure 1(a2)). IDT fingers with a 25 µm width and spacing, corresponding a resonance frequency of ≈40 MHz, were fabricated on another flat LN substrate via common photolithography (MLA 150, Heidelberg Instruments, Heidelberg, Germany), 5 nm/450 nm Cr/Au sputtering deposition (Denton 18, Denton Vacuum, NJ, USA), and the lift‐off process (Figure 1(b1)). Room‐temperature LN bonding was performed after piranha cleaning and oxygen plasma surface activation (PS100, PVA TePla PS100, Corona, CA, USA). The bonded chip was clamped and heated up to 300 °C in an oven (HTCR 6/28, Carbolite, Hope Valley, UK) to enhance the bonding strength (Figure 1(c)). The bonding strength is stronger than both the acoustic pressure and the capillary pressure, at least ≈1 MPa, preventing debonding when SAW was passed into the fluid inside the nanoslit.

##### Operation of the Device

A radio frequency (≈ 40 MHz) alternating current signal was generated from a signal generator (WF1967 multifunction generator, NF Corporation, Yokohama, Japan), amplified via a 5 W amplifier (5U1000, Amplifier Research Corp., Souderton, Pennsylvania, USA), and transmitted to IDT electrodes via custom connecting pogo pins. An oscilloscope (InfiniiVision 2000 X‐Series, Keysight Technologies, Santa Rosa, CA, USA) was used to measure the electrical properties (e.g., voltage, current, power) applied to the SAW device. Absorbers (Dragon Skin 10 Medium, Smooth‐On, Inc., Macungie, PA, USA) were attached at the edges of the LN chip to prevent reflected waves.

Ultra‐pure DI water was used to avoid clogging and introduced into the reservoirs illustrated in Figure 1b,c. Discrete droplets of the DI water appeared at the traps after evaporation; as the water evaporated, the fluid interface retracted into these trap regions to minimize surface energy. Typical droplet volumes were 200 fL, based on observations of the droplet diameter of diameter *d* ≈ 50 µm and a height *h* ≈ 100 nm.

The computation of the capillary pressures for a droplet from ambient to entrapped within the device at a trap and at a neck between two traps is provided in the next subsection.

Fluid motion was recorded via inverted microscope (IN480TC–FL–MF603, Amscope, Irvine, CA, USA) combined with cameras (D5300, Minato, Tokyo, Japan, and FASTCAM Mini UX100, Photron, Tokyo, Japan). Fluid motion, velocity, and fluorescence intensity were analyzed via custom image processing (MATLAB, Mathworks, Natick, Massachusetts, USA) code. For experiments in mixing, fluorescent 50‐nm particles (Fluorescein‐5‐isothiocyanate (FITC) dyed polystyrene particles, Polysciences, Inc., Warrington, PA, USA) at a rather high concentration of 3.64 × 10^11^ particles per µL were used to visualize the flow and ability to mix.

##### Statistical Analysis

For the results plotted in Figure 3, observations were made regarding the behavior of the droplet for a combination of the SAW activation time and input power. Uncertainty in these parameters was less than one percent. Forty‐three results are plotted in Figure 3 without interpretation, either representing droplet splitting, movement from trap to trap, or a failure to either split or moves. For the fitting of data to the expected linear relationship between the splitting ratio and applied energy in Figure 3b, based on the physics, the distribution of the splitting ratio to applied energy was first confirmed to be normal via the Shapiro–Wilk test before using linear regression for the fitting line. The coefficient of determination *R* ^2^ = 0.779, indicating a reasonably good fit. Further, to test for the expected monotonic increase in the splitting ratio versus applied energy, Spearman's rank‐order correlation coefficient was computed and found to be *r* = 0.901 with a 95% confidence interval from 0.744 to 0.964, a nearly perfect increasing monotonicity.

Regarding the droplet mixing results in Figure 5, the pixel intensity data relevant to the droplet region was extracted from the images in Figure 5a–f and an additional 56 similar images. Data associated with the walls of the trap and outside the droplet were excluded from consideration. The resulting data were found to be normally distributed over the range 0–1 (black to 100% green) via Shapiro–Wilk tests of the data per image and of all data across all images. The standard deviation (SD) of the normalized pixel intensities was then computed^[^ ^41^ ^]^ to quantify the state of mixing. Notably, both begin with a similar standard deviation at 1, reflecting the broad range of pixel intensities at this time. Spearman's rank correlation coefficients were *r* = −0.982 while SAW was on with a 95% confidence interval from −0.992 to −0.962, and *r* = −0.814 while the SAW was off with a 95% confidence interval from −0.909 to −0.640. All statistical analysis was performed using GraphPad Prism v9 (GraphPad Software Inc., San Diego, CA, USA).

##### Analysis

The outcome of the acoustic manipulation of a droplet in the nanoslit system depends upon the acoustic energy transmitted into the droplet. This energy is a product of the input power and the duration of the SAW as plotted in Figure 3, and the fraction of the actual acoustic energy taken up by the droplet from the SAW‐carrying LN substrate. The behavior of the SAW‐manipulated droplet was divided into three regimes, failure, splitting, and moving to the next trap.

The surface energy of the droplet when at rest in a trap, *E*_0_ is calculated first. The trapped droplet was assumed to have a circular cylindrical shape with a known radius, *r*_0_, and height, *h*. Thus, the surface energy is given as *E*_0_ = *C*_0_ *h*γ = 2π*r*_0_ *h*γ.

This may be compared to the surface energy of the droplet when its right end reaches the middle and narrowest region of the neck. This is the free surface energy the droplet must possess to almost—but not quite—pass a portion of it to the next trap, *E*_1_, representing the boundary (see Figure 3) between failure (1) and splitting (2).

With more input acoustic energy, the droplet should be able to travel further into the neck, deforming more and passing at least part of it into the neck widening from its midpoint. At the point where the droplet is symmetrically squeezed (50:50) about the neck's midpoint is considered next. This is the definition of the boundary between regions 2 and 3, splitting and moving completely to the next trap. At this point, the droplet should have a surface energy of *E*_2_.

Finally, the input acoustic energy required to completely move the droplet to the next trap may be defined. This is actually only slightly more than the energy required to place the droplet at the midpoint of the neck, due to the induction of excess kinetic energy in the droplet that propels it past the neck. This increment is small, much less than, say, *E*_2_ − *E*_1_, because the hydrodynamic time is one to two orders of magnitude greater than the time required to pass additional acoustic energy into the system to entirely move the droplet to the next trap (region 3). Furthermore, once the droplet passes the narrowest point of the neck, the droplet will begin to return to a circular cylindrical shape. The energy it recovers from the reduction in its free surface energy produces additional kinetic energy sufficient to carry it on to the next trap.

For this analysis, the shape of the droplet is assumed to be a right circular cylinder when in the trap, a somewhat complex egg cylinder shape—a Hügelschäffer's ovoid^[^ ^43^ ^]^—when between the trap and neck, and an elliptical cylinder when trapped symmetrically in the neck. When symmetrically placed in the neck, one could make an improved assumption that the droplet is a Cassini (biconcave) oval cylinder, at very substantial increase in analysis complexity^[^ ^44^, ^45^ ^]^ as one seeks to find the circumference, without much improvement in the results.

The shape and surface energy of the droplet is first considered when it is symmetrically trapped at the neck between two traps. The surface energy of the drop is always *E*_*i*_ = *C*_*i*_ *h*γ, with *C*_*i*_ referring to the circumference of the droplet. When symmetrically trapped, this equation becomes *E*_2_ = *C*_2_ *h*γ, with *C*_2_ as the ellipse's circumference. Unfortunately, there is no closed‐form solution for the circumference of an ellipse, which is most often represented as an elliptical integral of the second kind. However, the eminent mathematician Ramanujan devised an accurate approximation for it,^[^ ^46^ ^]^

(1) $$C_{2}=4a\int_{0}^{\pi /2}\sqrt{1-e^{2}\sin^{2}\theta }d\theta \approx \pi \left(a+b\right)\left(1+\frac{3m}{10+\sqrt{4-3m}}\right),$$

where $m={\left(\frac{a-b}{a+b}\right)}^{2}$ is defined in terms of the semimajor and semiminor axes *a* and *b*. Here *b* = *d*/2, where *d* is the minimum width of the neck between the two traps. The semimajor length, *a*, may either be measured from experimental images or determined from knowledge of the droplet's original radius when it is a circular cylindrical shape in the trap. The droplet volume is *V* = π*abh* in the neck, and is $V=\pi hr_{0}^{2}$ in the trap, producing $a=V/\pi bh=r_{0}^{2}/b=2r_{0}^{2}/d$.

Next, the shape and surface energy of the droplet when its right end is located at the midpoint in the neck between the two traps may be considered. The surface energy may be estimated as *E*_1_ = *C*_1_ *h*γ, with *C*_1_ representing the circumference of an asymmetric ellipse that has the same semimajor and semiminor axis lengths as the droplet, but “egg” shaped, formally known as Hügelschäffer's ovoid. The following equation was employed for this purpose,^[^ ^43^, ^47^ ^]^

(2) $${\left(\frac{x}{a}\right)}^{2}+{\left(\frac{y}{b}e^{cx}\right)}^{2}=1,$$

with *c* = 0.35 to match the asymmetric shape of the observed droplet in this state. The circumference of this shape is given by

(3) $$C_{1}=2\int_{-a}^{a}\;\sqrt{{\left(1-\frac{x^{2}}{a^{2}}\right)}^{-1}b^{2}e^{-2cx}{\left(\frac{cx^{2}}{a^{2}}-\frac{x}{a^{2}}-c\right)}^{2}+1}\;dx,$$

and there is no known approximate solution to this equation. It may, however, be solved numerically without much difficulty.

From these results, the minimum energy required to transport the droplet to the neck but failing to transport any of the droplet to the next trap might be estimated. This is *E*_1_ − *E*_0_; the droplet would return to the original trap afterward. Similarly, the energy required to symmetrically place the droplet at the center of the neck in order to split it equally among the two traps was then at least *E*_2_ − *E*_0_. Before considering what was necessary to completely move the droplet to the next trap, the details of the input into the device required to deliver these required changes in the surface energy needs to be determined.

One may begin by assuming the acoustic energy is uniformly distributed across the width of the IDT's aperture, *w*, and that the aperture is larger than the droplet diameter, *w* > 2*r*_0_. Along the propagation direction, it was assumed the SAW was weakly attenuated as defined from an attenuation length α^−1^. Within this area, (*w*)(α^−1^), the acoustic power may be written^[^ ^39^ ^]^ as $\eta (1-\frac{1}{e})W$, determining the width and profile of the acoustic field, where η ≈ 0.15 is the electromechanical coupling coefficient of 127.86° Y‐rotated, X‐propagating LN,^[^ ^48^ ^]^ e is Euler's number, and *W* is the applied electrical power into the SAW device. Because of the assumption of a uniformly distributed acoustic power across the width of the SAW device's aperture, we may determine the acoustic energy transmitted into the droplet, *E*_ac_, by considering the droplet's surface area in contact with the SAW‐driven LN substrate,

(4) $$E_{\text{ac}}=\frac{A}{w\alpha^{-1}}\eta \left(1-\frac{1}{e}\right)Wt,$$

where *t* is the activation time of the SAW. By setting *E*_ac_ = *E*_*i*_ − *E*_0_ with *i* ∈ {1, 2} and noting every term in Equation (4) is known, except for the activation time *t* and applied SAW power *W*, we may solve for the product of these two parameters, *Wt*, to produce

(5) $$Wt=\frac{w\alpha^{-1}}{A\eta \left(1-\frac{1}{e}\right)}\left(C_{i}-C_{0}\right)h\gamma$$

to determine the input power and activation time required to almost transport the droplet out of the trap (*C*_1_ − *C*_0_) or split it 50:50 between the two traps (*C*_2_ − *C*_0_).

These results were used to produce the solid and dashed lines in Figure 3 and are independent of the experimental results, with the exception of the droplet diameter at the start of each experiment. They appear to be strongly correlated with the experimental observations.

Beyond the 50:50 splitting of the droplet defined by the dashed line in Figure 3, there still was the possibility of splitting a droplet such that the amount traveling to the next trap was between 50–100%, leaving 0–50% to return to the original trap. We now consider how an excess in acoustic energy, *E*_ac_ > *E*_2_ − *E*_0_ could lead to this outcome.

Suppose there is an acoustic energy *Wt*_0_ that precisely overcomes the surface energy of the droplet from its minimum *E*_0_ in the trap to its maximum *E*_2_ in the neck. In other words, set *E*_ac_ = *kWt*_0_ = *E*_2_ − *E*_0_, where $k=\frac{A}{w\alpha^{-1}}\eta \left(1-\frac{1}{e}\right)$, and recall the details of the acoustic energy in Equation (5). The droplet would end up symmetrically trapped in the neck, eventually splitting due to the favorable decrease in surface energy as the droplet halves drew themselves away from the center of the neck.

Now suppose the activation time is longer, *t*_1_ > *t*_0_, while maintaining the same applied input power, *W*. This increases the acoustic input energy by an amount corresponding to *kW*(*t*_1_ − *t*_0_). If this energy is assumed to pass to the kinetic energy of the droplet, *T*, then

(6) $$kW\left(t_{1}-t_{0}\right)=T=\frac{1}{2}mv^{2},$$

where *m* is the droplet mass and *v* is the droplet velocity when the SAW turns off at *t*_1_.

As the acoustic power does not change when the SAW is on, the droplet may be assumed to exhibit constant acceleration so that

(7) $$\frac{1}{2}v\left(t_{1}-t_{0}\right)=l/2,$$

where *l* is the length of the neck from one trap to the next. Combining Equations (5) and (7) produces

(8) $$kW\left(t_{1}-t_{0}\right)=\frac{1}{2}m{\left(\frac{l}{t_{1}-t_{0}}\right)}^{2}$$

and so

(9) $$t_{1}-t_{0}={\left(\frac{ml^{2}t_{0}}{2(C_{2}-C_{0})h\gamma }\right)}^{\frac{1}{3}}$$

as our result. While one could seek to determine the energy produced by such an increment in time, or aim to determine the velocity the droplet would need to have at the neck to ensure its complete passage to the next trap, it is most helpful to consider the time one must leave the SAW on beyond the time to trap the droplet 50:50 in the neck, *t*_1_ − *t*_0_. By substituting values from the experiments, one may estimate (*t*_1_ − *t*_0_) ∼ 10^−3^ s.

The time scale of the acoustic wave, 40 MHz SAW, is far less, on the order of 10^−6^ s. It is therefore possible to drive SAW for (*t*_1_ − *t*_0_) ∼ 10^−3^ s to split the droplet and put 50–100% of its volume in the next trap.

However, the hydrodynamics is much slower. The capillary time^[^ ^49^ ^]^ is on the order of 10^−2^ − 10^−1^ s, one to two orders of magnitude larger than *t*_1_ − *t*_0_. So though it is possible to control the SAW's timing, the hydrodynamics is too slow to allow controlled splitting of a droplet such that 50–100% of its volume is in the next trap.

To pinch off a droplet of less than 50%, the droplet initially moves at speed into the neck, responding to the quick onset of SAW that is faster than the capillary time. However, the SAW stops before the droplet progresses beyond the halfway point of the neck, and the slow hydrodynamics associated with the capillary time takes over to define the droplet's behavior. A smaller portion of the droplet already past the midpoint of the neck may be pinched off before the entire droplet returns to the original well.

However, to pinch off a droplet of more than 50%, the SAW must be applied for a longer period of time. Once the droplet begins to move and advances more than 50% of its volume through the neck, it is impossible to arrest the hydrodynamics in time to split a small portion, say, of the droplet from the trailing edge to leave it behind. By the time that trailing edge portion would be pinched off in capillary time, the entire droplet is already in the new trap.

The implication is that while a 50:50 split is possible with the application of energy equivalent to *E*_2_ − *E*_0_, moving the droplet completely to the next trap is simply a matter of applying slightly more energy. The dynamics takes care of the rest.

## Conflict of Interest

The authors declare no conflict of interest.

## Supporting information

Supporting Information

Supporting Information

Supporting Information

## Data Availability

The data that support the findings of this study are available from the corresponding author upon reasonable request.
