# Supplementary material for: Manipulation and Mixing of 200 Femtoliter Droplets in Nanofluidic Channels Using MHz‐Order Surface Acoustic Waves
Source: Adv Sci (Weinh). 2021 May 16;8(13):2100408. doi: 10.1002/advs.202100408 (PMC8261518; doi:10.1002/advs.202100408)
Supplement: Supplementary file 1 — Supporting Information [file ADVS-8-2100408-s002.pdf]

© 2021 Wiley-VCH GmbH

ADVANCED SCIENCE
Open Access

Supporting Information

for *Adv. Sci.*, DOI: 10.1002/advs.202100408

Manipulation and Mixing of 200 Femtoliter Droplets in Nanofluidic Channels Using MHz-Order Surface Acoustic Waves

*Naiqing Zhang, Amihai Horesh, and James Friend**

Supplementary Information for Manipulation and mixing of 200 femtoliter droplets in nanofluidic channels using MHz-order surface acoustic waves

Naiqing Zhang,[†] Amihai Horesh,[†] and James Friend[*,†,‡]

†*Medically Advanced Devices Lab, Center for Medical Devices, Department of Mechanical and Aerospace Engineering, Jacobs School of Engineering, 9500 Gilman Dr. MC0411, University of California San Diego, La Jolla, CA 92093, USA*

‡*Department of Surgery, School of Medicine, 9500 Gilman Dr. MC0411, University of California San Diego, La Jolla, CA 92093, USA*

E-mail: jfriend@eng.ucsd.edu

Supplementary Information

Videos: femtoliter droplet manipulation

A video illustrating the three regimes we observed in droplet manipulation in our device by using SAW is provided, entitled "SI - Femtoliter droplet manipulation using SAW.mp4". Another video is provided showing femtoliter droplet splitting, merging, and mixing in "SI - Femtoliter droplet merging and mixing using SAW.mp4".

Droplet manipulation regimes, linearly plotted

The behavior of a 200 fL droplet of fluid initially located in a trap in our device and exposed to SAW is provided in Suppl. Fig. S1(a) for DI water as a linear plot of the SAW applied power versus activation time, indicating the close correspondence of the theory and experimental results without the vagueness of a log-log plot as provided in the main text—but with less clarity as the data points are somewhat crowded here. As an example of another fluid, experimental results using isopropyl alcohol (IPA; CAS 67–63-0, SigmaAldrich, St. Louis, MO USA) with surface tension $\gamma = 22$ mN/m are plotted in Suppl. Fig. S1(b) against the analytical model showing a close correlation.

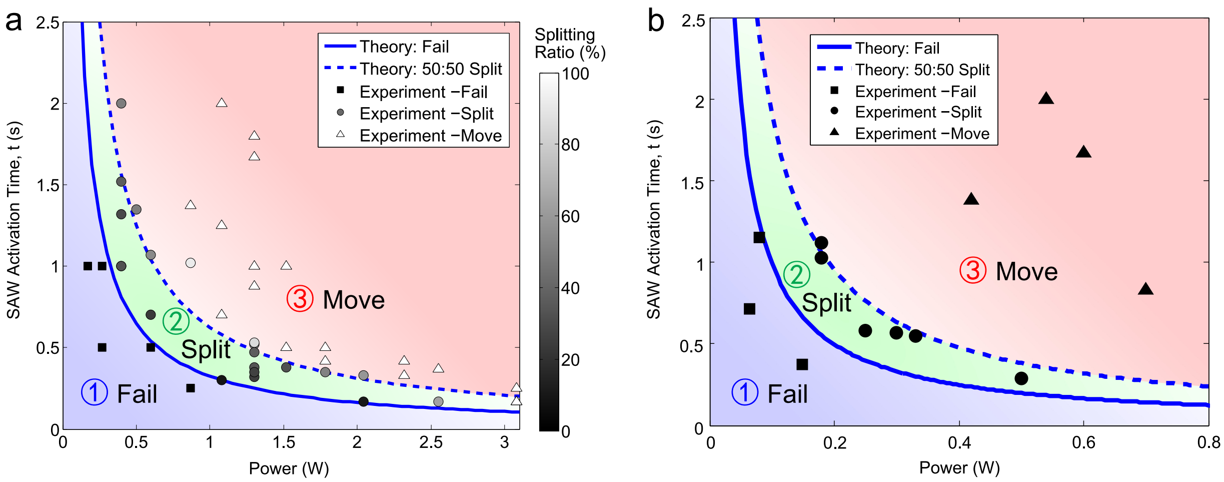

Figure S1: Analytical model and experimental results for droplet transport, plotted linearly as SAW activation time versus the input power. The results suggest three regimes in the nanoslit device between traps using (a) DI water and (b) IPA: failure, splitting, and complete moving of a droplet to the next well.

Image processing for droplet splitting ratio

The process to estimate the amount of fluid in droplets trapped in adjacent wells after splitting is illustrated in Suppl. Fig. S2. Thresholding here implies that the average pixel intensity was extracted from a given image, and those pixels possessing a greater value than the average were set to a value of one (white), while those less than the average were set to zero (black). Care was

taken to ensure no changes to intensity occurred during the experiments, prevented by locking the camera exposure and (electronic) shutter speed, and by documenting and maintaining identical lighting and optical conditions.

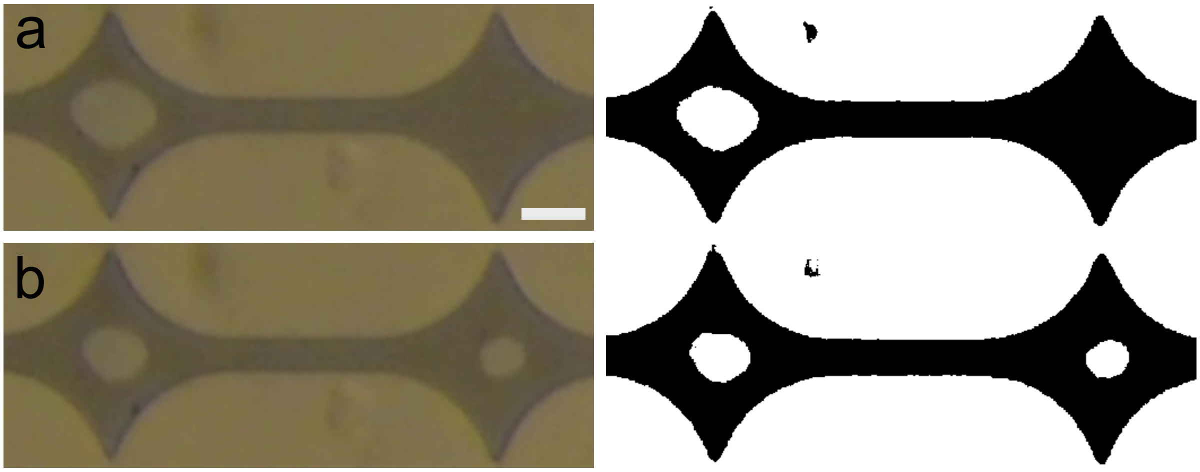

Figure S2: To determine the droplet splitting results, simple image processing was used. Bright-field images (left) were taken of the droplet(s) (a) before and (b) after droplet splitting. These were thresholded to produce black-and-white images (right), and the number of pixels present in the wells were counted and compared to determine the ratio of the droplet's volume in each trap. Scale bar: 40 μm.

Modeling the surface energy during manipulation

An illustrative figure to indicate what the models produce in representing the droplet shapes is provided in Suppl. Fig. S3. This information was used to produce the plots in the main manuscript that denote the splitting ratio versus input energy.

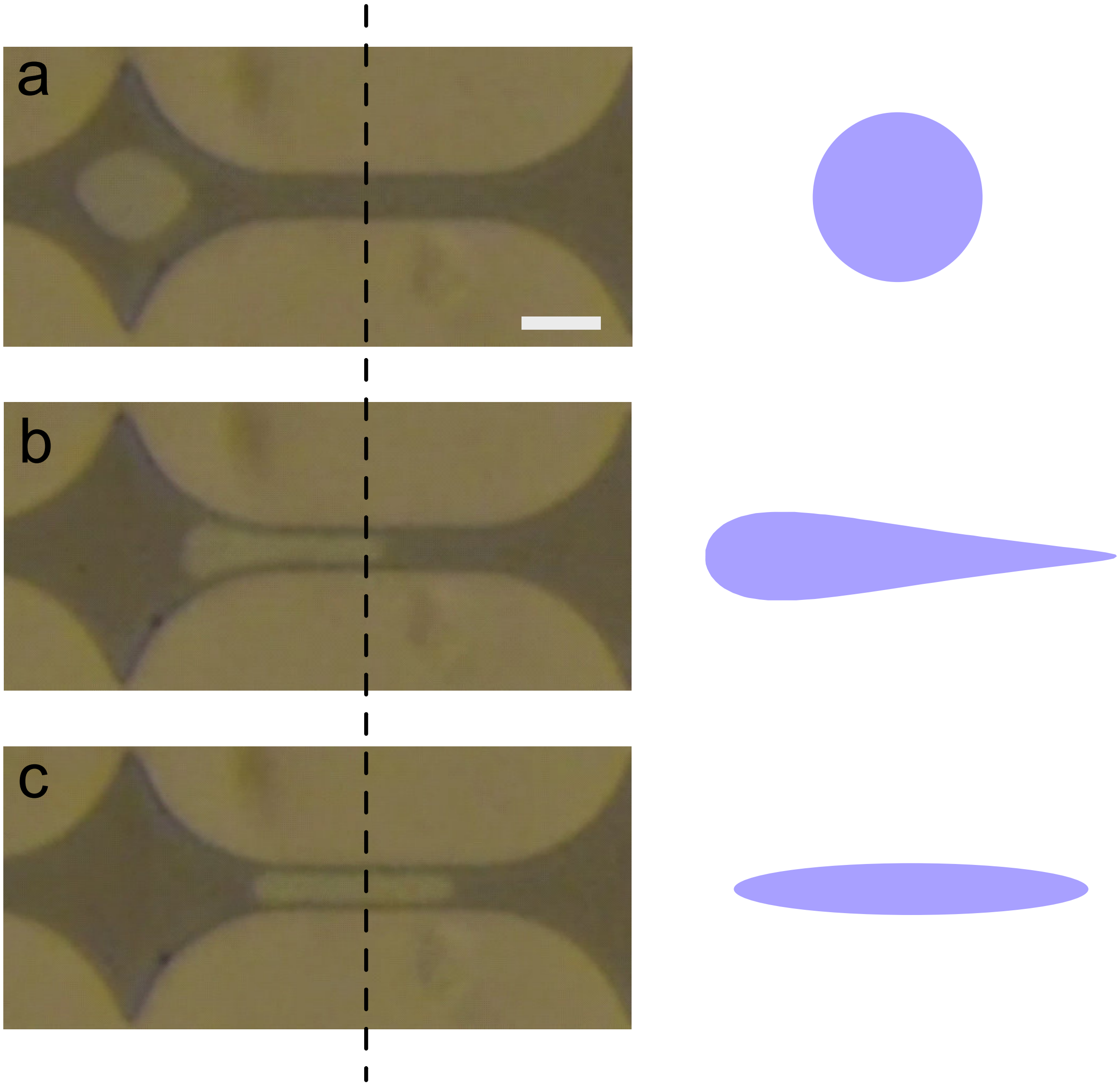

Figure S3: The droplet shape as it is transported from (a) its original position in the trap at left, to the (b) position corresponding with E_1 and C_1, where the droplet is at the boundary between failure (region 1 in Fig. 3) and splitting (region 2), and beyond to produce a 50:50 split between the two traps, represented by E_2 and C_2. The vertical dashed line is the midpoint of the neck between the two traps. The 50:50 split is also plotted as a dashed line in Fig. 3, main manuscript. On the left are images of the droplet for each state; on the right are corresponding plots of the droplet shape used in the analysis. Scale bar: 40 μm.
